# Third generation drug eluting stent (DES) with biodegradable polymer in diabetic patients: 5 years follow-up

**DOI:** 10.1186/s12933-017-0500-3

**Published:** 2017-02-10

**Authors:** Marcus Wiemer, Sinisa Stoikovic, Alexander Samol, Zisis Dimitriadis, Juan M. Ruiz-Nodar, Ralf Birkemeyer, Jacques Monsegu, Gérard Finet, David Hildick-Smith, Damras Tresukosol, Enrique Garcia Novo, Jacques J. Koolen, Emanuele Barbato, Gian Battista Danzi, on behalf of NOBORI 2 investigators

**Affiliations:** 1 Department of Cardiology and Intensive Care Medicine, Johannes Wesling University Hospital, Ruhr University Bochum, Hans-Nolte-Str. 1, 32429 Minden, Germany; 2 Clinic for Cardiology, Clinical Center of Serbia, School of Medicine, University of Belgrade, Belgrade, Serbia; 3 Department of Cardiology, Heart and Diabetes Center North Rhine-Westphalia, Ruhr University Bochum, Bad Oeynhausen, Germany; 4 Hospital General Universitario de Alicante, Universidad Miguel Hernández, Alicante, Spain; 5 Herzklinik Ulm, Ulm, Germany; 6 Groupe Hospitalier Mutualiste de Grenoble, Grenoble, France; 7 Hospital Cardiovasculaire Louis Pradel, Bron, France; 8 Sussex Cardiac Centre, Brighton and Sussex University Hospitals, Brighton, UK; 9 Siriraj Hospital, Bangkok, Thailand; 10 Hospital Guadalajara, Guadalajara, Spain; 11 St. Catharina Eindhoven, Eindhoven, The Netherlands; 12 Cardiovascular Research Center Aalst, OLV Hospital, Aalst, Belgium; 13 Ospedale Santa Corona, Pietra Ligure, Italy; 14 Department of Advanced Biomedical Sciences, University of Naples Federico II, Naples, Italy

## Abstract

**Objective:**

To report the long-term safety and efficacy data of a third generation drug eluting stent (DES) with biodegradable polymer in the complex patient population of diabetes mellitus after a follow-up period of 5 years.

**Background:**

After percutaneous coronary intervention patients with diabetes mellitus are under higher risk of death, restenosis and stent thrombosis (ST) compared to non-diabetic patients.

**Methods:**

In 126 centers worldwide 3067 patients were enrolled in the NOBORI 2 registry, 888 patients suffered from diabetes mellitus (DM), 213 of them (14%) being insulin dependent (IDDM). Five years follow-up has been completed in this study.

**Results:**

At 5 years, 89.3% of the patients were available for follow-up. The reported target lesion failure (TLF) rates at 5 years were 12.39% in DM group and 7.34% in non-DM group; (p < 0.0001). In the DM group, the TLF rate in patients with IDDM was significantly higher than in the non-IDDM subgroup (17.84 vs. 10.67%; p < 0.01). The rate of ST at 5 years was not different among diabetic versus non-diabetic patients or IDDM versus NIDDM. Only 10 (<0.4%) very late stent thrombotic events beyond 12 months occurred.

**Conclusions:**

The Nobori DES performed well in patients with DM. As expected patients with DM, particularly those with IDDM, had worse outcomes. However, the very low rate of very late stent thrombosis in IDDM patients might have significant clinical value in the treatment of these patients.

*Clinical trial registration* ISRCTN81649913; http://www.controlled-trials.com/isrctn/search.html?srch=81649913&sort=3&dir=desc&max=10

## Background

Upon introduction, drug-eluting stents (DES) with their anti-restenotic properties clearly paved the new path in interventional cardiology and directed future device improvements for the clinical benefit of patients [1]. Initial enthusiasm was suppressed by long-term follow-up data that depicted some late side effects, later proven to be mainly related to the unwanted persistence of only initially necessary anti-proliferative drug or durable polymer carrier [2, 3]. Since then, numerous stent design enhancements, known as “new stent generations” were marketed, succeeding not only to improve anti-restenotic efficacy but also to eliminate downsides of their predecessors [4]. Biodegradable polymer drug-eluting stents, were designed to provide polymer and drug free surroundings at the treatment site after early “vulnerable” restenotic period, thereby eliminating the potentially dangerous effects of persistent inflammatory stimulus [5]. With immediate efficacy evident, remote safety assumptions could not be proven until results of very long-term outcomes were available [6]. Recent developments in poly- and monomer technology demonstrated thromboresistance in blood-contact studies [7]. Potential biodegradable polymer technology advantages over durable polymer drug-eluting stents could be especially valuable in clinically complex patient subgroups, like patients with diabetes mellitus in whom results of percutaneous revascularization are known to be worse compared with non-diabetic population [8, 9]. In patients with diabetes, the use of DES was associated with a significant reduction of target lesion restenosis without an increase in adverse events compared to bare metal stents and the use of a polymer-free sotarolimus- and probutol eluting showed comparable long-term efficacy and safety as second-generation durable polymer zotarolimus-eluting stents [10, 11]. As previously reported, Nobori Biolimus A9™ eluting stent (Terumo corporation, Tokyo, Japan) with biodegradable polymer technology was associated with relatively low rates of adverse events in diabetic subgroup and no stent thrombosis up to 2 years of follow-up in insulin-dependent diabetes mellitus patients—a finding that demanded additional attention and investigation [12]. Therefore, the aim of this predefined sub-study was to further investigate long-term outcome up to 5 years of drug-eluting biodegradable polymer technology in high-risk population.

## Methods

### Patient population

Study design was previously reported in details [12, 13]. Briefly, the NOBORI 2 study was prospective, single-arm, multi-centre, worldwide registry designed to further validate safety and efficacy of the Nobori stent in real-world patients. Only exclusion criterion was patient inability or unwillingness to provide informed consent to participate. The studied population consisted of 3067 patients enrolled between April 2008 and March 2009 in a total of 126 centers across Europe and Asia. Through data entry in the electronic case report form, based on prior diagnosis, patients were automatically assigned to predefined analysis group of diabetic patients, and if they were on insulin therapy they were allocated to IDDM subgroup. Diabetes mellitus was diagnosed based on previous medical records of the patient and IDDM and NIDDM were differentiated based on presenting drug regimen of glucose lowering therapy. Age at diagnosis of DM was not recorded. No specific laboratory confirmation was requested for confirmation of DM. The study was conducted according to the Declaration of Helsinki, ISO 14155 and respecting all country-specific regulatory requirements. The protocol was reviewed and approved by the ethics committee of each participating hospital and all patients gave written informed consent.

### The Nobori Biolimus A9-eluting stent

The Nobori DES system that was used in the study was described in detail previously [14] and comprises four components: (1) the bare metal stent platform; (2) the delivery catheter; (3) the biodegradable drug carrier (polylactic acid); (4) an anti-proliferative substance, Biolimus A9™. Contrary to other DES, the drug polymer matrix is applied only abluminally (toward the vessel wall).

### Coronary stent procedure

Patients’ medication regimen, percutaneous access, lesion preparation, and stent implantations were performed according to hospital routine practice. The treatment of multiple target vessels and staged procedures were allowed. Peri-procedural dual antiplatelet and anticoagulation regimen were given at the discretion of the operators. A post-procedural electrocardiogram and the measurement of cardiac enzymes were recommended. Additional assessment of comorbidities was done using the Charlson comorbidity index [15].

### Patient follow-up

All patients were followed through hospital discharge and were scheduled for follow-up evaluations (hospital visit or telephone assessment) at 1, 6, and 12 months, and annually up to 5 years post-procedure. No mandatory angiographic follow-up was planned in this study. During the follow-up contacts, information about patients’ clinical condition, adverse events, hospitalizations, and changes to concomitant (cardiac and antiplatelet) medications were collected.

### Study management

Data were collected through standardized electronic case report forms (KIKA Medical, Boston, MA, USA). Noteworthy was to highlight a very high rate of data monitoring through online and on site check-ups. All major adverse cardiac events were assessed by independent clinical event committee. All baseline angiograms were analyzed by an independent core laboratory (CorExpert, Belgrade, Serbia).

### Study endpoints

The primary endpoint was Academic Research Consortium (ARC) defined, device oriented endpoint, a composite of cardiac death, myocardial infarction (MI) (Q-wave and non-Q-wave not clearly attributable to a non-target vessel) and target lesion revascularization (TLR), also known as target lesion failure (TLF). Secondary endpoints included: (1) TLF, (2) major adverse cardiac events (MACE) defined as cardiac death, MI, or any clinically driven target vessel revascularization (TVR), (3) death and MI, (4) TLR and TVR, (5) ARC defined, patient oriented composite endpoint (POCE) that included any death, any MI and any coronary revascularization (6) stent thrombosis according to ARC definitions [16]. All outcomes were evaluated at 12 months and yearly thereafter for 5 years.

### Statistical analysis

Data were presented as percentages and 95% confidence intervals for categorical variables, and means and standard deviations for continuous variables. All analyses were performed by an independent statistical office (SBD Analytics, Bekkevoort, Belgium) using SAS software, version 9.13 (SAS Institute Inc., Cary, NC, USA). All statistical tests were two-tailed with p < 0.05 considered to be statistically significant. Differences between IDDM and non-IDDM (NIDDM) patients were analyzed using Fisher’s exact test for binary variables, and Wilcoxon rank sum test for continuous variables.

## Results

### DM versus non-DM

**Fig. 1:**
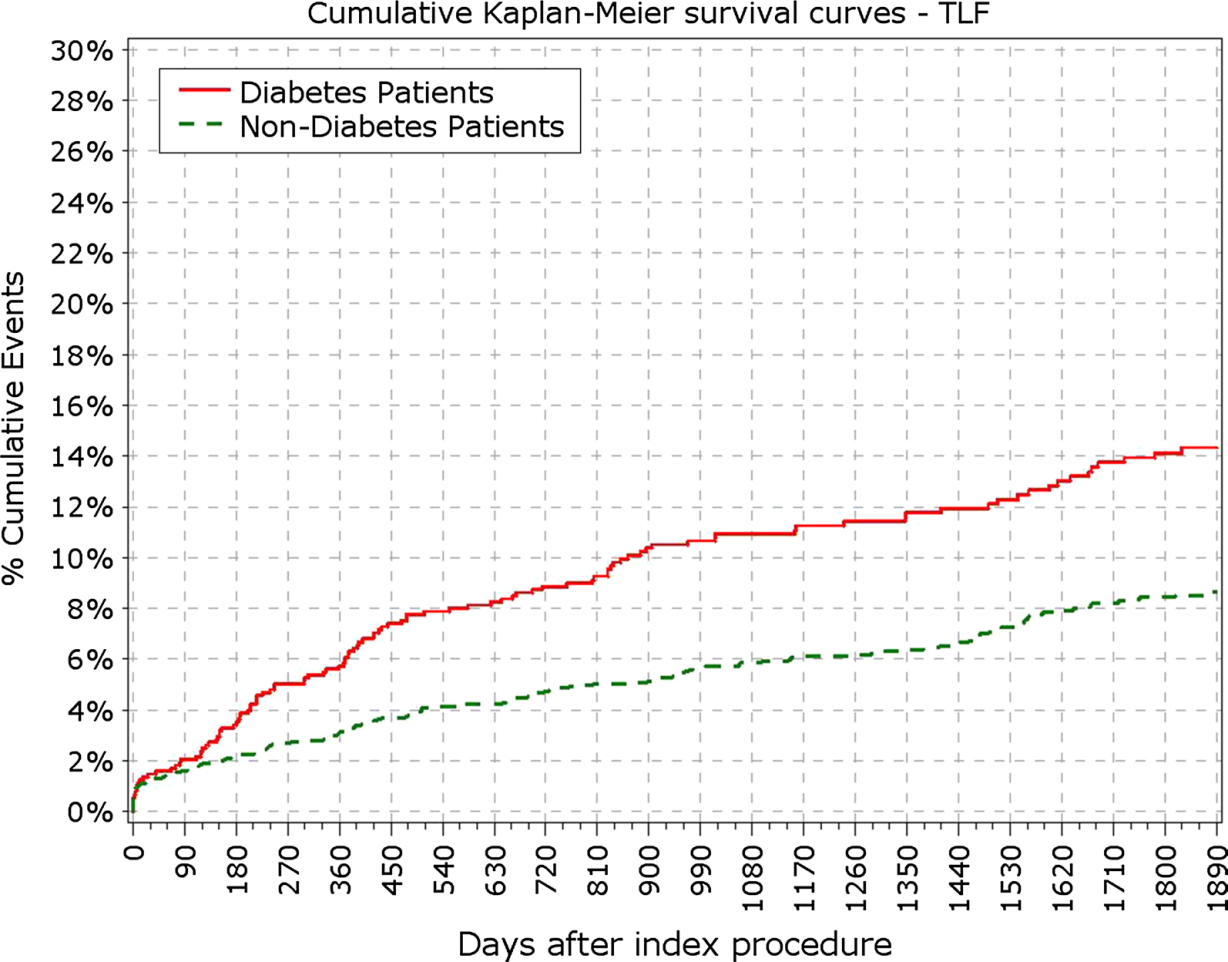
Primary endpoint: TLF incidence. *TLF* target lesion failure

**Fig. 2:**
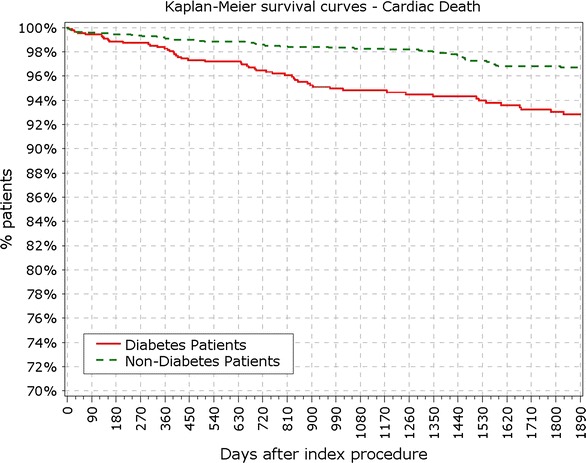
Secondary endpoint: survival from cardiac death

**Fig. 3:**
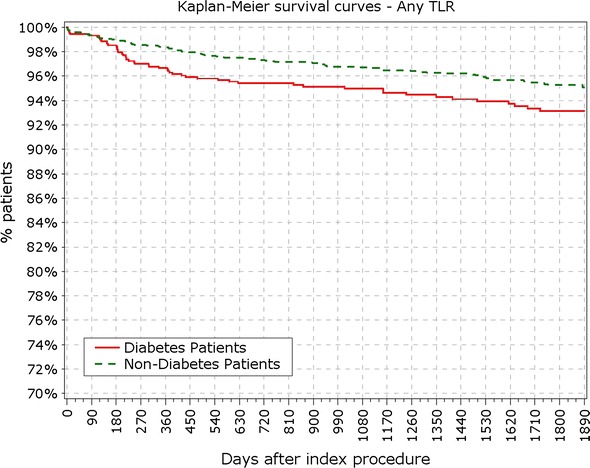
Secondary endpoint: survival from TLR. *TLR* target lesion revascularization

**Fig. 4:**
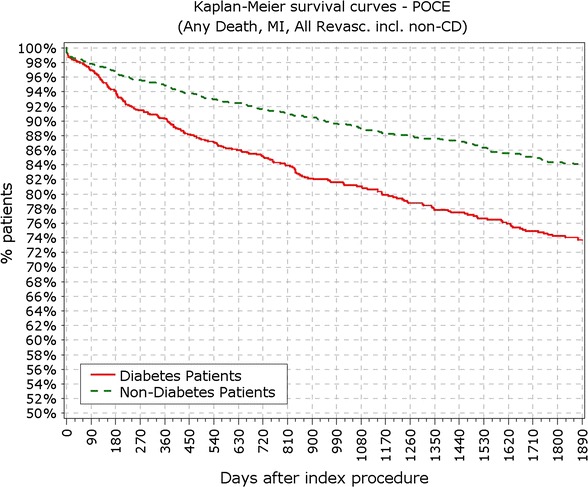
Survival from patient oriented composite endpoints. *POCE* patient oriented composite endpoints

The final study population included 3067 patients, among which 888 patients suffered from DM, with 213 of them (14%) being insulin-dependent DM (IDDM). Patient characteristics, baseline, procedural and quantitative coronary angiography analysis (QCA) were reported earlier and here are reported in Tables 1 and 2. BMI in patients with DM was significantly higher compared to patients without DM (28.9 vs. 27.2 kg/m^2^; p < 0.001). At 5 years, 89.3% of patients were available for follow-up. TLF rate in diabetic patients was significantly higher from year 1 (5.97 vs. 3.03%; p < 0.0001) up to end of the 5 years follow-up period (12.39 vs. 7.34%; p < 0.0001; Fig. 1; Table 3). As observed earlier this difference was driven mainly by cardiac death and TLR rates, while target vessel related MI rate did not differ in diabetic patients compared to non-diabetic (Figs. 2, 3). Rate of POCE was also significantly higher in DM group (22.86 vs. 13.72%; p < 0.0001) with half of the accumulated 5 years difference being generated within 12 months (11.60 vs. 6.79%; p < 0.0001; Fig. 4). Anginal status showed no differences between groups during study period with 87.87% of diabetic patients and 89.20% of non-diabetic patients being symptom free at the 5 years follow-up (p = 0.411; Table 3).

**Table 1:** Baseline characteristics

%	IDDM N = 213	Non-IDDMN N = 675	*P* (IDDM vs non-IDDM)	DM N = 888	Non-DM N = 2179	*P* (DM vs non-DM)
Age (years) (mean ± SD)	66.10 ± 10.13	66.56 ± 10.13	0.740	66.45 ± 10.12	63.53 ± 11.16	<0.001
Male sex	67.14	76.93	0.065	72.30	80.27	<0.001
Previous PCI	36.15	33.13	0.455	33.86	31.40	0.199
Previous CABG	13.62	8.06	0.021	9.40	8.57	0.481
Previous MI	33.01	32.83	1.000	32.87	33.27	0.864
Current smoker	16.94	18.12	0.826	17.85	28.66	<0.001
Previous smoker	31.15	37.73	0.115	36.20	33.97	0.269
Hypercholesterolemia	78.57	76.30	0.573	76.85	68.65	<0.001
Hypertension	86.38	79.70	0.034	81.31	64.03	<0.001
Family history of CAD	29.27	29.65	1.000	29.56	38.93	<0.001
Peripheral vascular disease	15.08	10.46	0.098	11.57	4.61	<0.001
Congestive heart failure	12.12	4.60	<0.001	6.39	2.89	<0.001
Charlson comorbidity index (mean ± SD)	2.61 ± 1.67	2.10 ± 1.27	<0.001	2.21 ± 1.39	0.84 ± 0.91	<0.001
Baseline anginal status						
Stable angina	46.23	44.66	0.693	45.03	46.12	0.603
Unstable angina	33.49	38.87	0.168	37.58	39.74	0.271
Silent ischemia	20.28	16.47	0.213	17.38	14.13	0.026
ACS	50.70	52.44	0.694	52.03	54.06	0.318
STEMI in ACS	8.3	16.1	0.042	14.3	15.5	0.59

*PCI* percutaneous coronary intervention, *CABG* coronary artery bypass graft, *CAD* coronary artery disease, *DM* diabetes mellitus, *IDDM* insulin-dependent diabetes mellitus, *ACS* acute coronary syndrome, *SD* standard deviation, *vs* versus, *MI* myocardial infarction, *N* number of patients

**Table 2:**
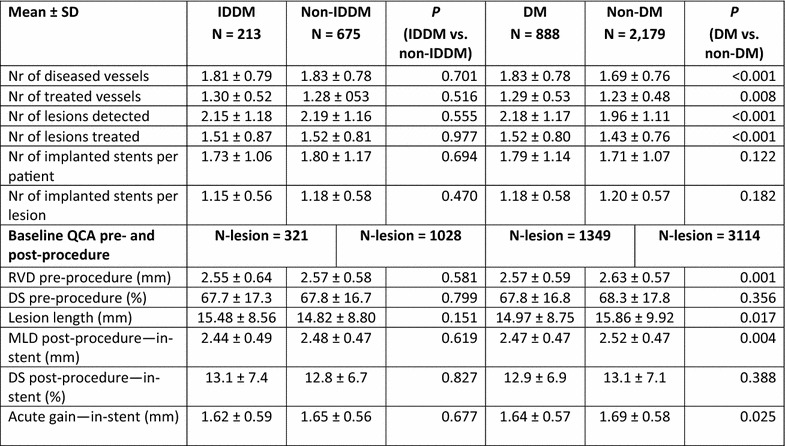
Procedural and QCA results

*QCA* qualitative comparative analysis, *SD* standard deviation, *N* number of patients, *DM* diabetes mellitus, *IDDM* insulin-dependent diabetes mellitus, *RVD* reference vessel diameter, *DS* diameter stenosis, *MLD* minimal luminal diameter, *Nr* number, *vs* versus

**Table 3:** Clinical outcomes at 1–5 years of follow-up

%	12 months			2 years			3 years			4 years			5 years		
	DM	NDM	P	DM	NDM	P	DM	NDM	P	DM	NDM	P	DM	NDM	P
Cardiac death	1.91	0.92	0.028	3.60	1.42	<.000	4.73	1.76	<.000	5.29	2.34	<.000	5.97	2.66	<.000
Non-cardiac death	0.90	0.41	0.111	2.14	1.10	0.040	3.38	1.7	0.006	4.84	2.11	<.000	5.41	2.57	<.000
Target vessel MI	1.80	1.33	0.324	2.82	1.88	0.130	3.15	2.16	0.121	3.49	2.25	0.060	3.83	2.52	0.057
TLR	3.49	1.65	0.003	4.39	2.71	0.023	4.73	3.12	0.032	5.41	3.63	0.028	5.86	4.08	0.036
TVR	4.62	2.57	0.004	2.14	1.65	0.369	7.21	5.05	0.025	8.33	5.78	0.012	2.82	2.25	0.365
MACE	7.8	4.5	<.000	11.2	6.4	<.000	13.1	7.9	<.000	14.5	9.0	<.000	15.8	10.1	<.000
POCE	11.60	6.79	<.000	15.2	8.44	<.000	18.24	10.6	<.000	21.06	12.25	<.000	22.86	13.72	<.000
TLF	5.97	3.03	<.000	8.67	4.77	<.000	10.25	5.6	<.000	11.15	6.52	<.000	12.39	7.34	<.000
Anginal status															
Stable angina	10.54	9.97	0.680	11.07	9.71	0.292	11.4	9.76	0.255	10.41	9.50	0.590	10.57	9.69	0.602
Unstable angina	0.86	1.08	0.684	1.03	0.91	0.827	0.15	0.71	0.127	0.41	0.96	0.373	1.37	0.96	0.454
Silent ischemia	0.74	1.52	0.102	0.90	1.42	0.346	1.06	1.54	0.440	0.41	0.48	1	0.20	0.15	1
No angina	87.87	87.43	0.802	87.00	87.96	0.519	87.39	87.99	0.674	88.78	89.06	0.865	87.87	89.20	0.411

*DM* diabetes mellitus, *NDM* patient without DM, *IDDM* insulin-dependent diabetes mellitus, *N* number of patients, *MI* myocardial infarction, *TLR* target lesion revascularization, *TVR* target vessel revascularization, *MACE* major adverse cardiovascular events, *POCE* patient-oriented composite endpoint, *TLF* target lesion failure, *vs* versus

### IDDM versus NIDDM

**Fig. 5:**
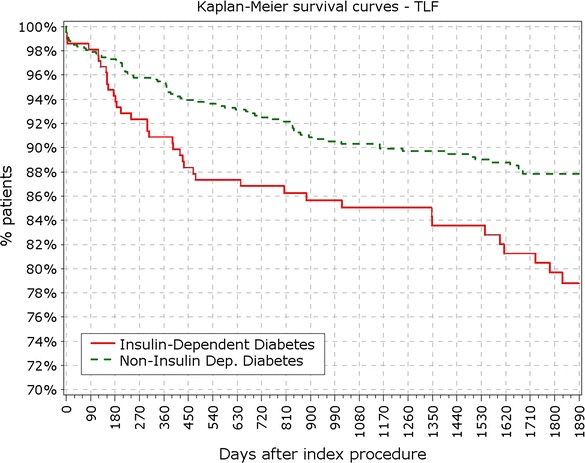
Survival from TLF in non-IDDM versus IDDM. *TLR* target lesion failure

**Fig. 6:**
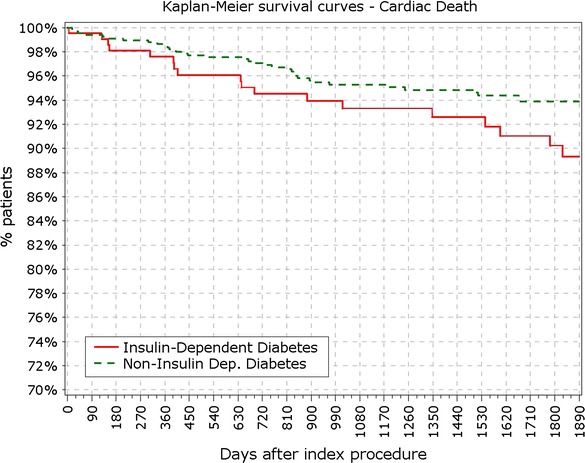
Survival from cardiac death in IDDM versus non-IDDM

**Fig. 7:**
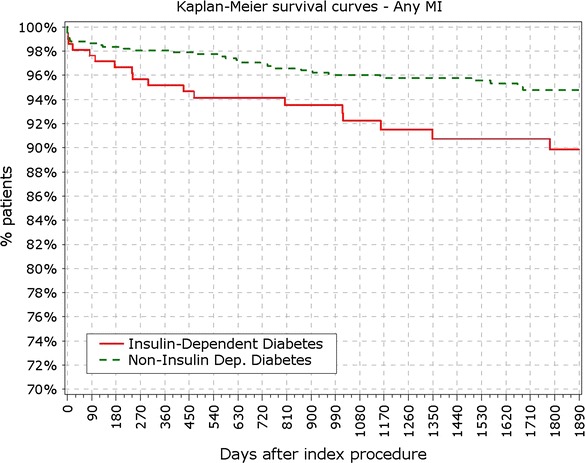
Survival from myocardial infarction in IDDM versus non-IDDM

**Fig. 8:**
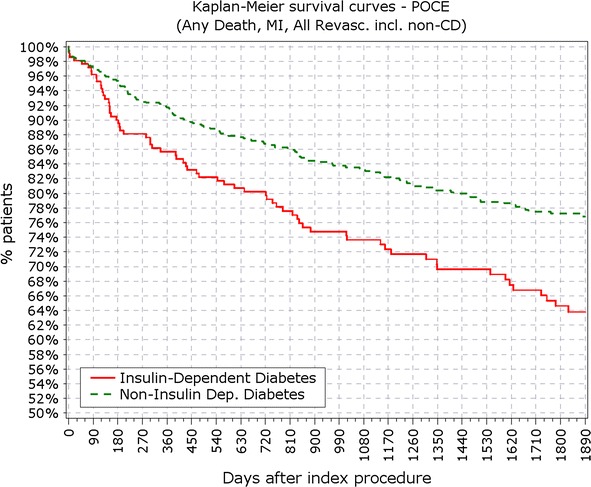
Survival from POCE in IDDM versus non-IDDM

BMI in patients with IDDM was significantly higher compared to patients with NIDDM (29.5 vs. 28.7 kg/m^2^; p < 0.001). Most of the difference in primary endpoints between non- and diabetic patients was driven by events occurring in IDDM patient subgroup (213 pts). Patients with IDDM had higher rate of TLF from 12 months (9.86 vs. 5.48%; p = 0.037) up to 5 years (17.84 vs. 10.67; p < 0.01; Fig. 5). Contrasting the findings between DM and non-DM, insulin dependence did not significantly increase the rate of TLR among DM patients not at 1 (5.63 vs. 3.11%; p = 0.09) or at the end of 5 years period (8.45 vs. 5.04%; p = 0.09). Rate of cardiac death and target vessel MI was not significantly increased in the IDDM group at 5 years or at any follow-up point, while rate of POCE showed early increment that persisted through the study period (Figs. 6, 7, 8).

### Stent thrombosis

Rate of study ST (definite and probable according to the ARC definitions) was not different among diabetic versus non-diabetic patients or IDDM versus NIDDM at any time point (Table 4). Most of the ST occurred within 30 days of implantation (19/34 ST; 55%) and only 10 (<0.4%) very late stent thrombotic events beyond 12 months were observed in whole study population.

**Table 4:** Stent thrombosis

Definite + probable study stent thrombosis up to	DM N = 888	Non-DM N = 2179	*P* (DM vs. non-DM)	IDDM N = 213	Non-IDDM N = 675	*P* (IDDM vs. non-IDDM)
30 days (early ST)	0.90% (8/888)	0.50% (11/2179)	0.2106	0.94% (2/213)	0.89% (6/675)	1
6 months	0.90% (8/888)	0.64% (14/2179)	0.480	0.94% (2/213)	0.89% (6/675)	1
12 months (late ST)	0.90% (8/888)	0.73% (16/2.179)	0.653	0.94% (2/213)	0.89% (6/675)	1
2 years	1.01% (9/888)	0.78% (17/2.179)	0.519	0.94% (2/213)	1.04% (7/675)	1
3 years	1.13% (10/888)	0.92% (20/2.179)	0.685	0.94% (2/213)	1.19% (8/675)	1
4 years	1.35% (12/888)	0.92% (20/2.179)	0.326	1.41% (3/213)	1.33% (9/675)	1
5 years	1.35% (12/888)	1.01% (22/2.179)	0.447	1.41% (3/213)	1.33% (9/675)	1

### Antiplatelet therapy

Mean loading dose of Clopidogrel was 415 mg in DM patients versus 442 mg in non-DM patients (p = 0.02) and 412 mg in IDDM patients versus 416 mg in NIDDM patients (p = 0.84). Dual antiplatelet therapy duration at 1 year was 77.4% in DM patients versus 71.4% in non-DM patients (p = 0.01), while for IDDM patients it was 77.3% compared to 77.4% in NIDDM patients (P = 1).

## Discussion

Present study shows long-term outcomes of biodegradable polymer drug eluting stent (BP-DES) treatment in highly unselected set of patients, especially in high-risk subgroup of patients with DM. Main findings of our analysis are: (1) Patients with DM have worse outcomes than non-diabetic patients with significantly higher rates of TLF, cardiac death and POCE throughout the whole study period; (2) Presence of insulin-dependent therapy among diabetic patients relates with more repeat revascularization events but not in higher rates of harder clinical endpoints, cardiac death and target vessel MI; (3) Cumulative rate of ST in whole population for the 5 years period was relatively low, especially rate of very-late ST; (4) Rate of stent thrombosis was similar between patients with and without DM, and insulin therapy did not increase the incidence of thrombotic events; Overall, our results confirm findings of previous reports that patients with DM, and especially those with IDDM, are at higher risk of adverse events following PCI [17]. Study results also demonstrated the overall very good performance of the BP-DES system in this high-risk patient population.

Clinical outcomes of Nobori stent implantation have been demonstrated in previous studies [12–14, 18]. This is the first study with a 5 years follow up specifically assessing its long-term performance in unselected group of patients with high-risk features for adverse prognosis like DM, especially IDDM.

Patients with DM compared to non-DM patients have less favorable outcomes in general, especially with percutaneous revascularization and with longer duration of follow-up [17]. Although the magnitude of restenosis reduction achieved with earlier DES platforms was impressive compared to BMS in most of the early randomized trials, this effect was not less evident in real world practice among patients with DM [19]. Differential clinical responses in patients with and without DM was documented even with new stent platforms highlighting the need for further opportunity to improve the treatment of CAD in patients with DM, particularly in those treated with insulin [20]. Meta-analyses showed that DES, compared to bare metal stents, were efficacious without compromising safety and assumed a potential highest benefit for patients with diabetes mellitus after treatment with everolimus eluting stents [11]. As expected, patients with DM had more adverse events in our analysis with higher TLR (5.8 vs. 4.1; p = 0.036), cardiac death (5.97 vs. 2.66%; p < 0.001) and composite endpoints MACE (15.8 vs. 10.1%; p < 0.001) and POCE (22.9 vs. 13.72%; p < 0.001).

Since results from long-term follow-up in diabetic patients treated with available biodegradable polymer stent platforms are lacking, it is justifiable to compare our findings to results of earlier DES platforms and durable polymer DES generations, demonstrating overall good performance of Nobori Biolimus A9 stent with low event rates in patients with or without DM (Table 4) [21–30]. Rate of MACE in DM group was lower than previously reported (15.8 vs. 17.0–40.5%) while rates of TLR (5.9 vs. 4.6–18.3%) cardiac death (5.9 vs. 2.4–15%) and MI (3.8 vs. 1.3–13.6%) also compared favorably with historical reports residing in lower edge of rates ranges. Very recently, the 5-years follow-up data of the diabetes subgroup of the ISAR Test 5 trial showed non-inferiority of a polymer-free sirolimus- and probucol-eluting stent compared to a second-generation durable polymer zotarolimus-eluting stent. Nevertheless, the rate of MACE, TLR, cardiac, and MI was higher in both arms of this study compared to our results. If these findings assume a superiority of BP-DES needs further investigation [10].

Long-term efficacy and safety of DES was subject of considerable debate with reports mainly from large-scale registries conflicting the excellent results of randomized trials focusing on immediate efficacy with shorter clinical evaluation periods. SCAAR study group drew attention of scientific community with results from large-scale registry showing increase in mortality in patients with DES compared with BMS before (HR 1.20) and after 6 months up to 3 years period (HR 1.32). High or prolonged antiproliferative drug delivery and persistent inflammatory propensities of durable polymers were heavily related with inadequate vessel healing, predisposing treated vessels to late adverse events [31–33]. Conceptually, design of biodegradable polymer stents were attractive solution since ultimately their long-term effects resemble BMS-like interaction with the vessel wall with less inflammatory stimulus. Recent reports showed that biodegradable polymers offer clear academic but equivocal clinical advantage. In large comparison, meta-analysis of 20.005 pts, treatment with BP-DES significantly reduced LLL and LST rates, without clear benefits on harder endpoints compared to durable polymer (DP)-DES [6]. High-risk population like STEMI patients also could benefit from BP-DES. In 497 patients with STEMI at 4 years, MACE was significantly reduced following treatment with BP-DES (hazard ratio [HR] 0.59, 95% CI 0.39–0.90; p = 0.01). Effect was driven by reduced TLR (HR 0.54, 95% CI 0.30–0.98; p = 0.04). Trends were also seen for cardiac death or MI (HR 0.63, 95% CI 0.37–1.05; p = 0.07) and definite or probable stent thrombosis (3.6 vs. 7.1%; HR 0.49, 95% CI 0.22–1.11; p = 0.09). Similar conclusions were drawn from pooled individual patient-level data from 3 randomized clinical trials [34] comparing biodegradable polymer DES with durable polymer (DP) DES. Clinical outcomes at 4 years were assessed. Out of 1094 patients with diabetes included in the analysis, 657 received BP-DES and 437 DP-DES. At 4 years, the incidence of the primary end point was similar with BP-DES versus DP-DES (HR 0.95, 95% CI 0.74–1.21, P = 0.67). But, rate of definite or probable stent thrombosis was significantly reduced in patients treated with BP-DES (HR 0.52, 95% CI 0.28–0.96, P = 0.04), and this difference was driven by significantly lower stent thrombosis rate with BP-DES after 1 and up to 4 years (HR 0.15, 95% CI 0.03–0.70, P = 0.02) eliminating the fear from late “catch-up” phenomenon with biodegradable polymers [35]. And indeed, serial optical coherence studies at 6, 12 and 24 months from implantation of BP-DES, Nobori Biolimus stent, did show that favorable features like, small gradual increase in neointimal thickness, with a nonsignificant decrease in the lumen area, lowering frequency of uncovered struts to almost none with very low percentage of detectable thrombi and peri-strut low-intensity area, could explain such low thrombotic risk beyond 1 year period. In addition, atherogenic neointima was not observed in the event-free OCT cohort [36]. When compared to Sirolimus eluting stent (SES) and BMS, BP-DES lies somewhere in-between according to recent OCT analysis. Biodegradable polymer Biolimus eluting stent showed a favorable coronary arterial response compared with SES, but different response with BMS at 5 years follow-up. The observed frequency of in-stent neoatherosclerosis within BP-DES was similar to BMS and tented to be lower than SES [37]. These obvious pathological and angiographically described potential advantages could explain low rates of ST occurring in 1.01, 1.35 and 1.41% in non-DM, DM and IDDM patients respectively up to 5 years with 24 (70%) occurring within 12 months. Rate of ST compared favorably to historical reports (0.8–10.2%). Results of trials with long term follow up after drug eluting stent implantation in patient with diabetes mellitus are summarized in Table 5.

**Table 5:** Overview of trials evaluating long-term outcomes of various DES types in diabetic subpopulation

(%)	Year	FU	Stent	N of pts DM	MACE	Cardiac death	MI	TVR	TLR	ST
Olesen et al.	2015	5 years	ZES	169	28.4	15	13.6	24.8	18.3	1
			SES	168	18.5	5	13.6	13.6	8.3	2.3
					p = 0.032	p < 0.01	p = ns	p < 0.01	p = 0.006	p = 0.02
Billinger et al.	2012	5 years	SES and PES combined	SES (503), PES (509)	25.9 vs. 19.2	11.4 vs. 4.3	6.5 vs. 6.8		14.4 vs. 14.1	6 vs. 4.6
					p = 0.02	p < 0.0001	p = ns		p = ns	p = ns
Onuma et al.	2011	5 years	BMS	112	53.6		11			
			SES	159	40.5		4.8	33.2		
			CABG	96	23.4		5.2	10.7		
					p < 0.01	p = ns	p = 0.04	p < 0.001		
Maeng et al.	2015	4 years	EES	108	20.4	8.3	1.9	12	5.6	0.9
			SES	105	23.8	4.8	7.6	16.2	9.5	1.9
					p = 0.55	p = 0.3	p = 0.067	p = 0.39	p = 0.28	p = 0.55
Simsek et al.	2013	3 years	EES	804	18	12	3.1	10.3	5.6	6.8
			SES	512	21.7	8.4	5.7	17.4	11.5	8.7
			PES	547	20.6	9.6	7.4	16.8	11.3	10.2
Stiermaier et al.	2014	5 years	SES	120	29.7	7.6	9.3	18.6	15.3	1.6
			PES	116	31.6	8.8	7.9	23.7	15.8	0.9
					p = 0.86	p = 0.94	p = 0.88	p = 0.44	p = 1.0	p = 1.0
Sinning et al.	2014	5 years	SES	95	36	15	8	NA	13	5
			MBS	95	52	13	9	NA	29	6
					p = 0.02	p = ns	p = ns		p = 0.003	p = ns
Sato et al.	2012	5 years	SES DM	85	27.1	2.4	4.7	15.3	9.4	3.5
			SES non-DM	112	16.1	2.7	0	14.3	8.9	0
					p = 0.06	p = 0.635	p = 0.827	p = 0.843	p = 0.886	p = 0.883
Park et al.	2012	5 years	SES	3101	17.5 vs. 12.3	1.8 vs. 2.0	6.8 vs. 4.2	9.6 vs. 6.9	8.8 vs. 6.0	2.3 vs. 2.2
			PES							
					p < 0.001	p = 0.623	p < 0.001	p < 0.001	p < 0.001	p = 0.820
Jensen et al.	2015	5 years	EES	194	17	8.2	2.6		4.6	2 pts
			SES	196	26.5	10.2	5.6		10.2	5 pts
Vardi et al.	2013	5 years	ZES	268	17.7	3.7	1.3	17.2	11.2	1.2
			SES and PES	268	26.6	7	5.1	20	12	0.8
					p = 0.012	p = 0.06	p = 0.011	p = ns	p = ns	p = ns

### Study limitations

Our study has several limitations. First, the diagnosis of DM was performed only on the bases of patients medical history without any confirmatory tests, potentially leading to lower incidence. Additionally, the diagnosis of diabetes was self reported and uncontrolled. Thus, the “non-DM” group could contain patients with unreported diabetes. Furthermore, NOBORI 2 is the non-randomized registry and the comparison with other DES is limited to historical data. Under-reporting of adverse events during follow-up is also possible. However, this registry is based on close online and on-site source data monitoring and relatively high follow-up compliance rate, we can assume that adverse event non-reporting can be considered as a matter of exception.

## Conclusion

 This analysis of the 5-years outcomes suggests that the Nobori biodegradable polymer DES is a suitable treatment option for overall but especially for high risk population subset like DM patients with clinical outcomes and safety profile that compare favourably to different DES platforms.

## Abbreviations

ARC: Academic Research Consortium
BMI: body mass index
BMS: bare metal stent
BP-DES: biodegradable polymer drug eluting stent
DES: drug eluting stent
DM: diabetes mellitus
DP-DES: durable polymer drug eluting stent
IDDM: insulin dependent diabetes mellitus
MACE: major adverse cardiovascular event
MI: myocardial infarction
NIDDM: non-insulin dependent diabetes mellitus
POCE: patient-oriented composite endpoint
SES: sirolimus eluting stent
ST: stent thrombosis
TLF: target lesion failure
TVR: target vessel revascularization
