# Erratum to: Third generation drug eluting stent (DES) with biodegradable polymer in diabetic patients: 5 years follow-up

**DOI:** 10.1186/s12933-017-0541-7

**Published:** 2017-05-04

**Authors:** Marcus Wiemer, Sinisa Stojkovic, Alexander Samol, Zisis Dimitriadis, Juan M. Ruiz-Nodar, Ralf Birkemeyer, Jacques Monsegu, Gérard Finet, David Hildick-Smith, Damras Tresukosol, Enrique Garcia Novo, Jacques J. Koolen, Emanuele Barbato, Gian Battista Danzi, on behalf of NOBORI 2 investigators

**Affiliations:** 1 Department of Cardiology and Intensive Care Medicine, Johannes Wesling University Hospital, Ruhr University Bochum, Hans-Nolte-Str. 1, 32429 Minden, Germany; 2 Clinic for Cardiology, Clinical Center of Serbia, School of Medicine, University of Belgrade, Belgrade, Serbia; 3 Department of Cardiology, Heart and Diabetes Center North Rhine-Westphalia, Ruhr University Bochum, Bad Oeynhausen, Germany; 4 Hospital General Universitario de Alicante, Universidad Miguel Hernández, Alicante, Spain; 5 Herzklinik Ulm, Ulm, Germany; 6 Groupe Hospitalier Mutualiste de Grenoble, Grenoble, France; 7 Hospital Cardiovasculaire Louis Pradel, Bron, France; 8 Sussex Cardiac Centre, Brighton and Sussex University Hospitals, Brighton, UK; 9 Siriraj Hospital, Bangkok, Thailand; 10 Hospital Guadalajara, Guadalajara, Spain; 11 St. Catharina Eindhoven, Eindhoven, The Netherlands; 12 Cardiovascular Research Center Aalst, OLV Hospital, Aalst, Belgium; 13 Ospedale Santa Corona, Pietra Ligure, Italy; 14 Department of Advanced Biomedical Sciences, University of Naples Federico II, Naples, Italy

## Erratum to: Cardiovasc Diabetol (2017) 16:23 DOI 10.1186/s12933-017-0500-3

After publication of the original article [1], it came to the authors’ attention that there was a typo within the author list. The family name of Sinisa Stojkovic was incorrectly spelled ‘Stoikovic’. The author’s name appears in its correct form in this erratum.
